# Quantiferon-TB Gold: Performance for Ruling out Active Tuberculosis in HIV-Infected Adults with High CD4 Count in Côte d'Ivoire, West Africa

**DOI:** 10.1371/journal.pone.0107245

**Published:** 2014-10-16

**Authors:** Christine Danel, Mathieu Kabran, André Inwoley, Anani Badje, Jean Louis Herrmann, Raoul Moh, Jérôme Lecarrou, Delphine Gabillard, Jean Baptiste Ntakpe, Nina Deschamps, Eric Ouattara, Christian Perronne, Serge Eholie, Xavier Anglaret

**Affiliations:** 1 INSERM, Centre INSERM U897, Bordeaux, France; 2 PAC-CI Program, CHU Treichville, Abidjan, Côte d'Ivoire; 3 ISPED, Bordeaux University, Bordeaux, France; 4 CeDReS, CHU Treichville, Abidjan, Côte d'Ivoire; 5 Service de Bactériologie, Hôpital Raymond Poincaré, Assistance Publique-Hôpitaux de Paris, Garches, France; 6 Département de Maladies Infectieuses et Tropicales, Hôpital Raymond Poincaré, Assistance Publique-Hôpitaux de Paris, Garches, France; 7 EA3647, Université de Versailles St Quentin en Yvelines, Montigny le Bretonneux, France; 8 Service des Maladies Infectieuses et Tropicales, CHU Treichville, Abidjan, Côte d'Ivoire; National Institute for Infectious Diseases (L. Spallanzani), Italy

## Abstract

**Objective:**

To assess the performance of QuantiFERON-TB Gold In-Tube (QFT-GIT) test for active tuberculosis (TB) in HIV adults, and its variation over time in patients on antiretroviral therapy (ART) and/or isoniazide preventive therapy (IPT).

**Methods:**

Transversal study and cohort nested in the Temprano ANRS 12136 randomized controlled trial assessing benefits of initiating ART earlier than currently recommended by World Health Organization, with or without a 6-month IPT. Performance of QFT-GIT for detecting active TB at baseline in the first 50% participants, and 12-month incidence of conversion/reversion in the first 25% participants were assessed. QFT-GIT threshold for positivity was 0.35 IU/ml.

**Results:**

Among the 975 first participants (median baseline CD4 count 383/mm^3^, positive QFT-GIT test 35%), 2.7% had active TB at baseline. QFT-GIT sensitivity, specificity, positive and negative predictive value for active TB were 88.0%, 66.6%, 6.5% and 99.5%. For the 444 patients with a second test at 12 months, rates for conversion and reversion were 9.3% and 14%. Reversion was more frequent in patients without ART and younger patients. IPT and early ART were not associated with reversion/conversion.

**Conclusion:**

A negative QFT-GIT could rule out active TB in HIV-infected adults not severely immunosuppressed, thus avoiding repeated TB testing and accelerating diagnosis and care for other diseases.

**Trial Registration:**

ClinicalTrials.gov NCT00495651.

## Introduction

Tuberculosis (TB) disease and HIV infection are two epidemics with strong interactions in sub-Saharan Africa [1]. Two interventions can reduce the incidence of TB disease in HIV infected patients: antiretroviral treatment (ART) [2], and isoniazid preventive therapy (IPT) [3], [4], [5], [6], [7] The latter has been shown to be more effective in patients with proven asymptomatic TB infection [5], [7].

The purified protein derivative (PPD) skin test used to detect TB infection has several limitations. First, it utilizes a relatively non specific and complex mixture of antigens, thus it can be positive in patients who have been vaccinated with the BCG vaccine or exposed to non-tuberculous mycobacteria [8]. Second, it can be negative in HIV-infected patients with low CD4 count, even if they are infected with TB [9]. Finally, results may vary depending on the experience of readers [10], [11], and the additional visit for reading the test after two days may be a challenge [12].

During the last decade, *Interferon-Gamma Release Assays* (IGRAs) have been developed as an alternative to the PPD test. IGRAs are diagnostic tests using a peptide cocktail that simulates antigens associated with *Mycobacterium tuberculosis* infection. The *In vitro* production of gamma interferon is then detected by an Enzyme-Linked Immunosorbent Assay (QuantiFERON-TB Gold in-tube [QTF-GIT],) [13] or by the Enzyme-Linked Immunospot (T-SPOT TB) [14] IGRAs are more sensitive and more specific than the PPD test for the diagnosis of TB infection [15]–[18], and especially useful in BCG-vaccinated populations. They do not require an additional visit for reading and are more reproducible than the PPD test, but they are more costly (54 Euros *vs.* 2.16 Euros) [19].

At present, the role of IGRAs compared to PPD test, in clinical practice, remains unclear. In industrialized countries, situations in which an IGRA may be preferred to a PPD test include testing persons from groups that have low rates of returning to have the PPD read, and persons who have received BCG as a vaccine or for cancer therapy [20]. In middle and low income countries, WHO recently concluded that there was insufficient data and low quality evidence on the performance of IGRAs to recommend their use [21]. There is a strong consensus that additional research is needed on the value and limitations of IGRAs in situations related to tuberculosis control and clinical practice [20], [21].

In 2008 we launched the Temprano trial, a randomized controlled trial of early ART, concomitantly or not with a 6-month IPT, in HIV-infected adults with high CD4 counts in Abidjan, Côte d'Ivoire. An ancillary study of Temprano was done in the first 50% participants, with three objectives: (i) to assess the performance of QTF- GIT for the diagnosis of active TB at inclusion in the trial; (ii) to estimate the 12-month cumulative incidence of conversion and reversion of QTF-GIT in patients with negative or positive QTF-GIT at baseline; (iii) to estimate the 30-month incidence of active TB in patients with negative and positive QTF-GIT at baseline, overall and by trial intervention, i.e. early ART and/or IPT.

We report here the results of objectives (i) and (ii) of the Temprano QTF-GIT ancillary study.

## Methods

### Patients

Temprano is a multicentric randomized open-label trial to assess the benefits and risks of two interventions in patients with high CD4 count: early ART initiation, and 6-month IPT. The trial began in March 2008 in 9 clinical centres in Abidjan, Côte d'Ivoire. The trial protocol was approved by the institutional review board of the French Research Agency on AIDS and viral hepatitis (ANRS, Paris) and by the Côte d'Ivoire National Ethics Committee. It has been registered on clinicaltrials.gov under the identifier NCT00495651. The protocol of the trial is also avalaible at http://mereva.net/temprano.

Here we report the results of an ancillary study of Temprano, in which a group of participants in the trial had a QTF-GIT test at baseline and at Month-12.

### Temprano trial design

The trial inclusion criteria are: HIV-1 or HIV 1+2 dual seropositivity; age ≥18 years; signed informed consent; absence of ongoing active TB; no ongoing pregnancy; a CD4 count ≤800/mm^3^ and no CD4 count–based or clinical stage-based indication to start ART immediately, according to the most recent WHO guidelines. The latter criterion evolves in line with WHO guidelines updates. Once enrolled, patients are randomized into four arms: immediate ART, deferred ART, immediate ART plus 6-month IPT, and deferred ART plus 6-month IPT. Immediate ART consists of starting ART at inclusion (Day-0), irrespective of patients' CD4 count and clinical stage. Deferred ART consists of starting ART at any time during follow-up, as soon as WHO clinical and immunological criteria for starting ART are met [22]. IPT consists of a six-month course of isoniazid (300 mg once a day), starting at the Month-1 visit and stopping at Month-7 visit. The trial sample size was calculated at 2076 participants, based on the assumption of cumulative proportion of severe morbidity at Month 30 at 10% and a 40% reduction of the incidence of severe morbidity in the early ART.

Each participant is followed 30 months. The trial main outcome is the occurrence of a new episode of severe morbidity, including AIDS-defining diseases, non-AIDS defining severe bacterial diseases, non-AIDS defining cancers, and any event leading to death. In the present sub study, the assumption for the sample size calculation was a proportion of active Tuberculosis at 30 months follow up at 4% in patients with positive QTF TB test at inclusion and 1% in negative ones. Based on the software N Query with the α risk at 5% and the 1-β at 80%. 860 patients are needed to show a difference between the 2 groups. With the assumption of 1,7% lost to follow up, we needed 922 patients.

### Temprano trial procedures

On Day-0, blood samples are collected for blood cell count, CD4 cell count (Trucount technique, FACSCalibur), serum transaminases, serum creatinine, serum glucose, and plasma HIV-1 RNA (real-time PCR, detectability 300 copies/mL). Patients had a chest radiograph at inclusion. Patients randomized to immediate ART start treatment on Day-1. The first-line regimen is preferably a fixed dose combination of tenofovir disoproxil fumarate 300 mg and emtricitabine 250 mg (Truvada, 1 tablet once a day) plus efavirenz 600 mg (Stocrin, 1 tablet once a day). Patients with contra-indication to efavirenz were given preferably Truvada plus lopinavir/ritonavir 400/100 mg (Kaletra, 2 *heat*-stable tablets twice a day). At the end of the Day-0 visit, patients are asked to show up for trial scheduled visits at Day-8, Month-1, Month-2, Month-3, and every 3 months thereafter. Standardised questionnaires are used to record baseline and monthly characteristics. Transport, consultations, investigations, hospitalizations and drugs are free-of-charge.

Patients have access to their study clinic at any time. Patients with signs or symptoms consistent with TB (fever, cough, weight loss, hemoptysis, lymphadenopathy) are investigated with symptom-guided specimen examination for the presence of mycobacteria, and other appropriate tests. Samples are processed for acid fast bacilli smear (AFB) after auramine staining and then cultured on both BacT/Alert 3D liquid culture system (bioMérieux, Durham, NC, USA) and Loweinsten-Jensen (LJ) medium. Positive BacT/Alert vials are analyzed by Ziehl-Neelsen staining to confirm the presence of AFB. For all positive cultures, the differentiation between *M. tuberculosis* complex strains and mycobacteria other than tuberculosis (MOTT) is done by means of morphological characteristics and the radiometric NAP (p-nitro-alpha-acetylamino-beta-hydroxypropiophenone) inhibition test (Becton Dickinson, Sparks, MD, USA). Drug susceptibility testing (DST) is performed by using a broth based assay, the Bactec S.I.R.E Drug kit (Becton Dickinson Microbiology Systems, Sparks, MD).

Each episode of morbidity is reviewed by an independent event documentation committee, using standardized criteria [23]. If a given episode of morbidity, including TB, is documented after Day-0, the event documentation committee evaluates whether the episode could be proved to have started before Day-0 or not. TB infection is defined as a positive QTF test without clinical signs of evolutive infection (fever, cough, hemoptysis, weight loss, lymphadenopathy). Latent TB has the same definition as TB infection. Probable Active TB is defined as clinical signs suggesting active TB, and evidence of acid-fast bacilli (or typical appearance in immunofluorescence) in sputum sample and other extra-pulmonary liquids and tissues; or typical granulomatous appearance of the histological sample. It is classified as Definitive TB if *Mycobacterium tuberculosis*, *M. bovis* or *M. africanum* is identified in sputum samples or other extra-pulmonary fluids or tissue cultures, or presence of typical caseous appearance of the histological sample. Patients are considered to have: (i) a prevalent episode of active TB if the first objective sign of TB recorded in the trial case report form or in the patient's clinical centre file was proved to exist prior to or at Day-0; (ii) an incident episode of active TB if no objective sign of TB could be clearly proved to exist prior to or at Day-0. Whenever a doubt exists, the episode is conservatively labelled “incident”.

### Additional procedures

For the present study, QFT-GIT tests were done at Day-0 (n = 976) and at Month-12 (n = 430) in the first 50% and 25% participants in the trial. Venous blood was drawn into three 1 ml tubes, one containing heparin alone (negative control), another containing mitogen (positive control), and a third tube coated with TB antigens, early secreted antigen target 6 (ESAT-6), culture filtrate protein 10 (CFP-10), and TB7.7 (Rv2654) peptides.

After mixing, all tubes were incubated at 37°C for 16–24 hours within two hours of collection. Plasma was then separated by centrifugation and stored at −20°C before performing ELISA.

The test consists of a negative control (‘Nil’ well, whole blood without antigens or mitogen), a positive control (‘Mitogen’ well, whole blood stimulated with the mitogen phytohaemagglutinin) and a sample well (‘TB Antigen’ well, whole blood stimulated with ESAT-6, CFP-10 and TB7.7). QFT-GIT values are based on the amount of IFN-γ released in response to the antigens. The IFN-γ level of the Nil well is considered the background value and is subtracted from the mitogen and antigen-stimulated well values.

Base on the manufacturer's guidelines, the test is considered: positive if Nil ≤8.0 IU/ml, TB Antigen minus Nil ≥0.35 IU/ml and ≥25% of Nil value; negative if Nil ≤8.0 IU/ml, TB Antigen minus Nil <0.35 IU/ml and Mitogen minus Nil ≥0.50 IU/ml, or if Nil ≤8.0 IU/ml, TB Antigen minus Nil ≥0.35 IU/ml and <25% of Nil value, and Mitogen minus Nil ≥0.5 0IU/ml; and indeterminate in any other cases. For the present study, we chose TB Antigen minus Nil ≥0.35 IU/mL as the threshold for positivity, we consider the result as indeterminate if Mitogen minus Nil ≤0.45UI/ml. Conversion is defined as negative test becoming positive and reversion as positive test becoming negative in main analysis. We then varied this threshold in sensitivity analysis.

### Analysis

Fisher exact or Chi-square tests were used to compare patients' baseline characteristics by QFT-GIT result. We estimated the specificity (Sp), sensibility (Se), negative predictive value (NPV) and positive predictive value (PPV) of the QFT-GIT test at baseline for the diagnosis of prevalent active TB, *ie.* the presence of an episode of active TB being proved to pre-exist at Day-0. The method used to estimate the confidence intervals was the standard Wald asymptotic confidence limits for the risks based on the normal approximation to the binomial distribution.

The 12-month cumulative incidence of conversion and reversion were estimated with 95% confidence intervals. Logistic regression models were used to analyze the association of patients' baseline and follow-up characteristics with conversion and reversion. Variables with a p-value<0.25 in univariable analysis were included in multivariable analysis. Analyses were performed with SAS software, version 9.2 (SAS institute Inc. Cary, North Caroline, USA).

## Results

### Baseline (Day-0) and follow-up characteristics

**Figure 1:**
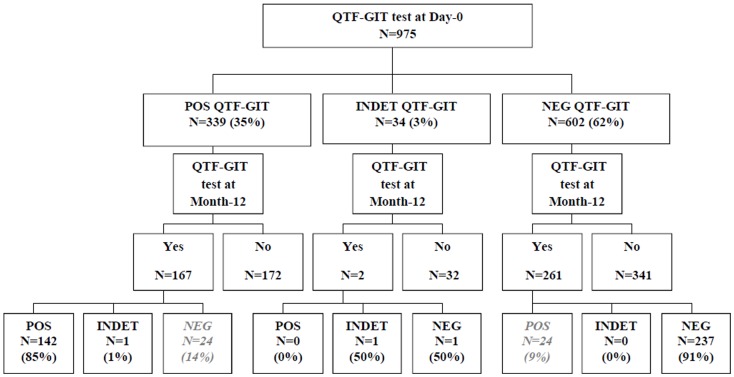
Flow chart. POS: Positive QuantiFERON TB Gold in-tube test; NEG: Positive QuantiFERON TB Gold in-tube test; INDET: Indeterminate QuantiFERON TB Gold in-tube test.

The first 1000 patients were included in the Temprano Trial between March 18, 2008 and August 21^st^, 2009. Of these 1000 patients, 975 had a QFT-GIT test at Day-0 (figure 1S). Seventy seven percent were women, their median age was 35 years, their median CD4 count 383/mm^3^, and their median plasma HIV-1 viral load 4.7 log_10_ copies/ml (table S1).

Twenty five patients (2.7%) were diagnosed with a prevalent episode of active TB at Day-0, including 16 with pulmonary TB, 6 with extrapulmonary TB (pleuritis, n = 3; peripheral lymph nodes, n = 3) and 3 with both pulmonary and extra-pulmonary TB (pleuritis, n = 1, femoral osteitis plus abdominal lymph nodes, n = 1, peripheral lymph node n = 1)(Table S3).

**Figure 2:**
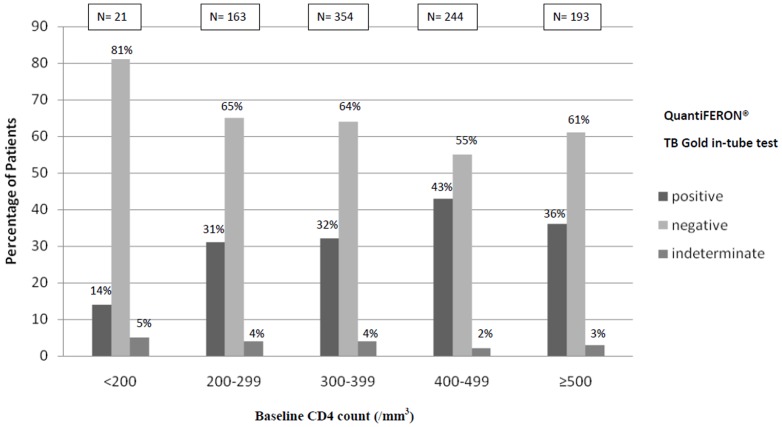
QuantiFERON TB Gold in-tube test results at Day-0, by CD4 count category. ^a^ positive: Nil ≤8.0 IU/ml, TB Antigen minus Nil ≥0.35 IU/ml and ≥25% of Nil value; ^b^ negative: Nil ≤8.0 IU/ml, TB Antigen minus Nil <0.35 IU/ml and Mitogen minus Nil ≥0.45; or Nil ≤8.0 IU/ml, TB Antigen minus Nil ≥0.35 IU/ml and <25% of Nil value, and Mitogen minus Nil ≥0.45; ^c^ indeterminate: Nil tube >8.0 IU/ml, or Nil tube ≤8.0 IU/ml and TB Antigen minus Nil <0.35 IU/ml and Mitogen minus Nil <0.45; or Nil tube ≥8.0 IU/ml and TB Antigen minus Nil ≥0.35 IU/ml and <25% of Nil value, and Mitogen minus Nil <0.45.

Of the 975 participants, 339 (35%) had a positive QFT-GIT test, 602 (62%) had a negative test, and 34 (3%) had indeterminate results. Patients with positive tests had lower viral load and higher CD4 counts than those with negative or indeterminate tests (table S1). The percentage of patients with positive tests increased with increasing CD4 counts, from 14% in patients with <200 CD4 to 43% in those with 400–500 CD4/mm^3^ (p = 0.01)(figure 2S).

QFT-GIT test positivity was significantly more frequent in patients with ongoing active TB at Day-0. Among the 25 patients with ongoing active TB, 22 (88%) had a positive QFT-GIT test. Among the 950 patients with no active TB at inclusion, 317 (33%) had a QFT-GIT positive test.

### Performance of The QuantiFERON TB test at baseline (Day-0)

Using the manufacturer's definition, the performance of the QFT-GIT test for the diagnosis of active TB disease at Day-0 were: sensitivity 88.0% [95%CI 75.3–100]; specificity 66.6% [95%CI 63.6–69.6]; positive predictive value 6.5% [95%CI 3.9–9.1]; and negative predictive value 99.5% [95%CI 99.0–100].

When stratified for baseline CD4 count, we found respectively: sensitivity, specificity, positive predictive value, and negative predictive value of 87.5% [95%CI 64.63–100]; 72.2% [95%CI 66.7–76.8]; 6.48% [95%CI 1.84–11.1] and 99.6% [95%CI 98.9–100] when CD4 count was below 350/mm^3^; and 88.2% [95%CI 72.9–100]; 63.2% [95%CI: 59.3–67.1]; 6. 49% [95%CI 3.32–9.67] and 99.5% [95%CI 98.7–100] when CD4 count was above 350/mm^3^.

In sensitivity analysis, when the threshold for positivity was increased from 0.35 UI/ml to 10 UI/ml, sensitivity decreased to 44.0%, specificity increased to 90.5%, positive predictive value increased to 10.9% and negative predictive value decreased to 98.4% (table S2).

**Figure 3:**
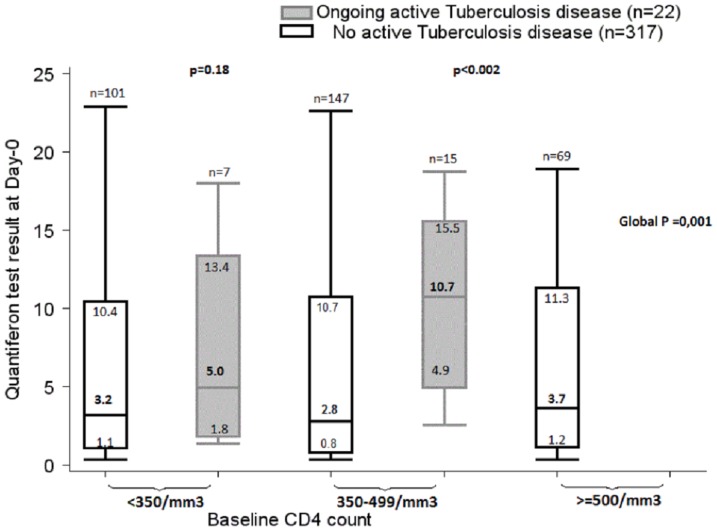
Distribution of QuantiFERON TB Gold in-tube test results at Day-0, by CD4 count category in patients with positive tests and with or without active tuberculosis. Box plots show median value, interquartile range, and range. *p*: Kruskal-Wallis test.

Among patients with a positive QFT-GIT test, the median value was 3.2 UI/ml (Interquartile range [IQR] 0.9–10.7) in those with no ongoing active TB and 10.0 UI/ml (IQR 4.9–13.4) in those with ongoing active TB (p = 0.001). When stratifying by baseline CD4 count, the median QFT- GIT test value was higher in patients with ongoing active TB than in those with no TB. This difference reached significance in patients with 350–500 CD4/mm^3^ (Figure 3S).

### Conversion and reversion between baseline (Day-0) and Month-12

Of the 975 patients with a QTF-GIT test at baseline, the first 430 had a repeated QTF-GIT test at Month-12. The characteristics at Day-0 of the 430 patients who had a 12-Month test did not differ significantly from that of the 545 patients who did not (data not shown).

The median (IQR) CD4 cells count calculated at baseline in the ART patients group (patients who start ART during the 12 first months) and the No ART patient group (patients who do not start ART during the same period) was 383/mm^3^ (325; 465) and 406/mm^3^ (352; 522) groups respectively (p = 0,01). In addition, the percentage of patients included in the study, with CD4 cells count less than 500/mm^3^ at baseline was 66% and 34% in the ART patients group and in the no ART patients groups respectively (p = 0,04).

Between baseline and Month-12, 169 (39%) of the 430 patients with repeated QTF-GIT test received a 6-month IPT and 276 (64%) started ART (all with tenofovir- emtricitabine, in association with: efavirenz in 74%, lopinavir/ritonavir in 24%, and zidovudine in 2%) at any time between baseline and month-12. 104 receive IPT and ART. Overall, the median difference between the Day-0 CD4 count and the Month-12 CD4 count was +69/mm^3^ (IQR −43;+231). Nine (2%) patients had a prevalent episode of TB at Day-0, and 11 (3%) developed an incident episode of active TB between Day-0 and Month-12 (pulmonary 5; extrapulmonary TB 4, Mixed 2).

Of the 167 patients with a positive QTF-GIT test at Day-0, 24 (14%) had a reversion at Month-12. For these 24 patients, the median baseline QFT-GIT value was 1.1 UI/ml (IQR; 0.6;3.0) 10 received IPT among them.

Of the 261 patients with a negative QTF-GIT test at Day-0, 24 (9%) had a conversion at Month-12. In these 24 patients with conversion, the median Month-12 QFT-G value was 1.0 UI/ml (IQR 0.6;10.5).

The percentage of conversion or reversion of the QuantiFERON TB Gold in-tube test between Day-0 and Month-12, varies according to different thresholds chosen (Table S4).

Of the 9 (2%) patients with an ongoing prevalent episode of active TB at Day-0, 8 had a positive QTF-GIT test at Day-0 (median value 8.5 UI/ml; IQR: 5.0;15.0); of these 8 patients, 7 had still a positive test at month-12 (median value 2.6 UI/ml; IQR: 1.0–11.2) and one had a negative test at Month-12. For the latter, the baseline test value was 1.36 UI/ml.

Among the 11 (3%) who developed a new incident episode of active TB between Day-0 and Month-12, 6 had a positive QTF-GIT test at Day-0 (median value 14.4 UI/ml; IQR: 11.4–18.7), all of them being still positive at Month-12 (median value 5.2 UI/ml; IQR: 2.3–23.6); and 5 had a negative QTF-GIT test at Day-0, of whom one had a positive test at Month-12 (13.1 UI/ml) and four had negative tests at Month-12.

In univariable analysis, no factors (among: IPT, ART, age, hemoglobin, viral load, WHO clinical stage, gender, HBs Ag, BMI, CD4 evolution, baseline CD4) were associated with conversion (Table S5). Age, ART and a CD4 decrease (≥1 cell/mm^3^) between baseline and Month-12 were associated with reversion. In multivariable analysis, reversion was significantly more frequent in patients who did not receive ART as compared to patients who started ART between Day-0 and Month-12 (OR = 4.8; IC 95%CI 1.8–12.6; p = 0.002), and in younger patients (<40 *versus.* ≥40 years old: OR 6.7, 95% CI 1.5–30.3; p = 0.01) (Table S6).

The median difference in CD4 count between baseline and Month-12 was +86/mm^3^ (IQR −38; +239) for the 143 patients with no reversion and −40/mm^3^ (IQR −130, +104) for the 24 patients with reversion.

## Discussion

We have highlighted several features in this population of HIV infected adults with high CD4 counts in Abidjan.

First, even if the sensitivity of QTF-GIT was better than that of TB smear [25], and similar to that of Xpert tests [28], [29], its specificity was far lower than that of both tests. As a result, in our population where 2.7% of people had active TB, its positive predictive value was always ≤10%, even when using high thresholds for positivity. This confirms that the test is of no relevant clinical use to confirm the diagnosis of TB. These data are similar to that previously reported by others [24]–[27].

Second, in our population, QTF-GIT had a negative predictive value of 99.5% for the diagnosis of active TB, even for patients with CD4 count below 350/mm^3^. This excellent negative predictive value suggests that QFT-GIT could be a valuable tool to rule out the diagnosis of active TB in HIV-infected but not severely immunosuppressed patients in sub-Saharan Africa. In a recent meta-analysis of Xpert tests compared to smear microscopy in HIV positive patients [28], the mean negative predictive value of Xpert tests for active TB was 90.5%, a figure much lower and of much less interest for clinical use than that we found in our study.

In Saharan Africa, TB is frequently suspected, less frequently confirmed, and even less frequently ruled out. A reliable test to rule out TB would be especially useful in HIV- infected patients, in whom suspicion of TB entails repeated and time consuming investigations. Ruling out TB with a highly predictive test could pave the way for clinicians to focus on differential diagnoses and for the patients to receive timely appropriate treatment for diseases other than TB. It could also reduce unwarranted empiric TB treatment that involves added risks to the patient, such as: drugs interactions between ART and anti-TB treatment; necessity to switch ART regimens in patients who receive antiretroviral drugs that are contraindicated in combination with rifampicin; drug toxicity; and poor adherence due to the burden of pills, which in turn increases the risk of selecting resistance to HIV drugs [26].

The main limitations for widespread use of this test in developing countries are its cost, and the technical facilities required [19], [21].

In our study the proportion of indeterminate or negative QTF-GIT tests was higher in people with CD4 counts below 200/mm^3^ [27], and the rate of reversion at 12 months was higher in those who had not started ART. These two findings suggest that immunodepression may lead to false-negative tests, thus making the interpretation of a negative test more difficult in patients with very low CD4 counts [24], [30], [31].

Third, we found high rates of QTF-GIT reversion or conversion at 12 months.

Our rate of reversion (14%) was of the same magnitude as that previously reported by others [32]–[34]. Reversion may be due to healing of TB infection linked to better immunity. In our study, the higher CD4 cells count at baseline in the No ART patient group might explain the higher reversion rate in patients not having started ART, and the association of younger age with reversion [35]. IPT was not significantly associated with QTF-GIT reversion, which is consistent with other recent reports [32], [35]–[37]. This could be due to: (i) persistent TB infection with latent bacilli; (ii) TB reinfection, in this immune deficient population living in a high TB prevalence setting; (iii) the interval between the end of IPT and the second QTF-GIT test being too short (5 months) to achieve reversion; and (iv) lack of power.

Our rate of conversion (9.2%) may be explained by three mechanisms: (i) new TB infections; Previously reported QFT-GIT conversion rates in HIV-negative health workers living in countries with high TB prevalence were of similar magnitude [38], [39], [40]; (ii) false negative tests becoming truly positive after immune restoration [41] However, in our study the absence of association between conversion and ART or CD4 gain is not in favor of this hypothesis; (iii) intra-individual variability of the test. [42], [43]. (iv) In our study, IPT was not associated with conversion, possibly due to lack of power.

The usefulness of QFT-GIT does not only rely on its negative predictive value for current TB infection. It may also be of value in predicting the risk of developing active TB in the future, thus allowing the identification of patients who may benefit the most from IPT. To our knowledge, no longitudinal studies have ever analyzed this long-term predictive aspect in HIV-infected patients in sub-Saharan Africa. This analysis will be done after the end of the Temprano trial, which is scheduled for December 2014.

In conclusion, our study suggests that QFT-GIT could have a role along with the other TB tests, TB smear and Xpert, to help clinicians manage patients with signs or symptoms of TB in sub-Saharan Africa. This is particularly helpful in HIV-infected patients in whom extra-pulmonary and smear negative forms are frequent. Even though the test does not help rule in active TB, [29], it has a very high negative predictive value and therefore could help rule out active TB in HIV infected people who are not severely immunosuppressed. This would be especially worthwhile if further studies confirm that the QFT-GIT negative predictive value for TB is higher than that of Xpert tests.

## Supporting Information

Table S1 **Patients baseline characteristics, according to QuantiFERON-TB Gold in-tube test results at Day-0.**
(DOCX)

Table S2 **Performance of QuantiFERON TB Gold in-tube test for the diagnosis of ongoing active Tuberculosis disease at Day-0.**
(DOCX)

Table S3 **Active Tuberculosis and QuantiFERON TB Gold in-tube results: details on the 25 active TB cases diagnosed at Day-0.**
(DOCX)

Table S4 **Conversion and reversion of the QuantiFERON TB Gold in-tube test between Day-0 and Month-12, according to different thresholds.**
(DOCX)

Table S5 **Factors associated with conversion, defined as QTF <0,35UI/ml at baseline and QTFTB test >0,35UI/ml at Month-12, univariable analysis.**
(DOCX)

Table S6 **Factors associated with reversion, defined as QTF >0,35UI/ml at baseline and QTFTB test <0,35UI/ml at Month-12, univariable and multivariable analysis.**
(DOCX)
