# Supplementary material for: Quantiferon-TB Gold: Performance for Ruling out Active Tuberculosis in HIV-Infected Adults with High CD4 Count in Côte d'Ivoire, West Africa
Source: PLoS One. 2014 Oct 16;9(10):e107245. doi: 10.1371/journal.pone.0107245 (PMC4199568; doi:10.1371/journal.pone.0107245)
Supplement: Table S1 — Patients baseline characteristics, according to QuantiFERON-TB Gold in-tube test results at Day-0. (DOCX) [file pone.0107245.s001.docx]

**Table 1S : Patients baseline characteristics, according to QuantiFERON^®^-TB Gold in-tube test results at Day-0**

|  | **Overall** | **QuantiFERON^®^-TB Gold at Day-0** | | | |
| --- | --- | --- | --- | --- | --- |
|  |  | **Positive ^a^** | **Négative ^b^** | **Indeterminate ^c^** | ***p*** |
| **Patients, n (%)** | 975 (100) | 339 (35) | 602 (62) | 34 (3) |  |
| **Women, n (%)** | 752 (77) | 259 (76) | 469 (78) | 24 (71) | *0.57* |
| **Age in years, median (IQR)** | 35 (30-42) | 36 (30-43) | 35 (30-42) | 40 (29-45) | *0.13* |
| **CD4 cells/mm^3^, median (IQR)** | 383 (317; 472) | 404 (330-484) | 374 (310-469) | 364 (305-437) | *0.03* |
| **Viral load, log_10_copies/ml, median (IQR)** | 4.7 (4.0-5.2) | 4.6 (4.0-5.2) | 4.7 (4.0-5.2) | 5.2 (4.4-5.7) | *0.02* |
| **WHO clinical stage, n (%)** |  |  |  |  | *<0.001* |
| Stage 1 | 626 (64) | 197 (58) | 411 (68) | 18 (53) |  |
| Stage 2 | 234 (24) | 90 (27) | 136 (23) | 8 (24) |  |
| Stage 3 | 107 (11) | 46 (14) | 54 (9) | 7 (21) |  |
| Stage 4 | 8 (1) | 6 (2) | 1 (0) | 1 (3) |  |
| **Positive serum HBs Ag, n (%)** | 97 (10) | 29 (9) | 65(11) | 3 (9) | *0.58* |
| **Serum transaminases > 2.5 x ULN, n (%)** | 4 (0) | 1 (0) | 3 (0) | 0 (0) | *1.00* |
| **Active Tuberculosis, n (%)*** | 25 (2.7) | 22 (6.8) | 1 (0.2) | 2 (5.6) | *<0.001* |
| Pulmonary | 16 | 14 | 1 | 1 |  |
| Extra pulmonary | 6 | 6 | 0 | 0 |  |
| Pulmonary and extra pulmonary | 3 | 2 | 0 | 1 |  |

**Footnotes to table 1:**

^a^ positive: Nil ≤ 8.0 IU/ml, TB Antigen minus Nil >0.35 IU/ml and>25% of Nil value;

^b^ negative: Nil ≤ 8.0 IU/ml, TB Antigen minus Nil <0.35 IU/ml and Mitogen minus Nil > 0.45, or if Nil ≤ 8.0 IU/ml, TB Antigen minus Nil >0.35 IU/ml and <25% of Nil value and Mitogen minus Nil > 0.45;

^c^ indeterminate: Nil tube > 8.0 IU/ml, or Nil tube ≤ 8.0 IU/ml and TB Antigen minus Nil <0.35 IU/ml and Mitogen minus Nil < 0.45, or Nil tube > 8.0 IU/ml and TB Antigen minus Nil >0.35 IU/ml and <25% of Nil value and Mitogen minus Nil < 0.45

n: number; %: percentage; IQR: interquartile range; viral load : plasma HIV-1 RNA; HBs Ag: Hepatitis B surface antigen; ULN: upper limit of normal; IU: international units

* see details on the 24 cases in technical appendix, table 1A.
