# Supplementary material for: Quantiferon-TB Gold: Performance for Ruling out Active Tuberculosis in HIV-Infected Adults with High CD4 Count in Côte d'Ivoire, West Africa
Source: PLoS One. 2014 Oct 16;9(10):e107245. doi: 10.1371/journal.pone.0107245 (PMC4199568; doi:10.1371/journal.pone.0107245)
Supplement: Table S2 — Performance of QuantiFERON TB Gold in-tube test for the diagnosis of ongoing active Tuberculosis disease at Day-0. (DOCX) [file pone.0107245.s002.docx]

**Table 2S : Performance of QuantiFERON^®^ TB Gold in-tube test for the diagnosis of ongoing active Tuberculosis disease at Day-0**

|  | Performance of the QuantiFERON^®^ TB Gold test  for the diagnosis of active Tuberculosis | | | | | | | |
| --- | --- | --- | --- | --- | --- | --- | --- | --- |
| QuantiFERON^®^ TB Gold test  Threshold of positivity (IU/ml) * | Sensitivity | | Specificity | | Positive predictive value | | Negative predictive value | |
|  | % (IC95%) | | % (IC95%) | | % (IC95%) | | % (IC95%) | |
| > 0.35 | 88.0 | (75.3-100) | 66.6 | (63.6-69.6) | 6.5 | (3.9-9.1) | 99.5 | (99.0-100) |
| > 1 | 88.0 | (75.3-100) | 76.0 | (73.3-78.7) | 8.8 | (5.3-12.3) | 99.6 | (99.1-100) |
| > 5 | 56.0 | (36.5-75.5) | 86.6 | (84.5-88.8) | 9.9 | (5.0-14.9) | 98.7 | (97.9-99.5) |
| > 7.5 | 52.0 | (32.4-71.6) | 88.8 | (86.8-90.8) | 10.9 | (5.3-16.5) | 98.6 | (97.8-99.4) |
| > 10 | 44.0 | (24.5-63.5) | 90.5 | (88.7-92.4) | 10.9 | (4.8-17.0) | 98.4 | (97.6-99.2) |

Footnotes to table 2:

IU/ml: international units per millimeter

* In patients with Nil ≤ 8.0 IU/ml and TB Antigen minus Nil >0.35 IU/ml and >25% of Nil value
