# Supplementary material for: Quantiferon-TB Gold: Performance for Ruling out Active Tuberculosis in HIV-Infected Adults with High CD4 Count in Côte d'Ivoire, West Africa
Source: PLoS One. 2014 Oct 16;9(10):e107245. doi: 10.1371/journal.pone.0107245 (PMC4199568; doi:10.1371/journal.pone.0107245)
Supplement: Table S3 — Active Tuberculosis and QuantiFERON TB Gold in-tube results: details on the 25 active TB cases diagnosed at Day-0. (DOCX) [file pone.0107245.s003.docx]

**Table 3S:** **Active Tuberculosis and QuantiFERON^®^ TB Gold results: details on the 25 active TB cases diagnosed at baseline**

| **Num** |  | **TB location** |  | **AFB*** | **Culture** | **ATB** | **QTF -G** | |  | **Baseline**  **CD4 count /mm*^3^* (%)** |
| --- | --- | --- | --- | --- | --- | --- | --- | --- | --- | --- |
|  |  |  |  | **(Pos/total)**  **Max AFB/field** |  |  | **Result** | **TB Antigen minus Nil (UI/ml)** |  |  |
| AB134 | D | Pulm + Mediastinal Lymph nodes |  | (0/6) | *Positive* | NA | Positive | 1,81 |  | 225 (15.2) |
| CF184 | P | Pulm + Pleura |  | (0/1) | Negative | - | Positive | 17,99 |  | 242 (15.2) |
| UC062 | D | Pulm, bone, peripheral Lymph nodes |  | (0/3) | *Positive* | S | Indeterminate | 0,1 |  | 269 (23.1) |
| AB036 | P | Mediastinal Lymph nodes |  | (0/3) | Negative | - | Positive | 9,95 |  | 453 (23.2) |
| HB065 | D | Peripheral Lymph nodes |  | (0/0) | *Positive* | S | Positive | 4,94 |  | 409 (17.6) |
| NS001 | P | Pleura |  | (0/1) | Negative | - | Positive | 10,86 |  | 456 (20.9) |
| ST025 | P | Pleura |  | (0/1) | Negative | - | Positive | 2,79 |  | 459 (9.9) |
| ST051 | P | Abdominal Lymph nodes |  | (0/5) | Negative | - | Positive | 4,46 |  | 233 (13.4) |
| UC102 | P | Pleura |  | (0/4) | Negative | - | Positive | 18,74 |  | 365 (23.5) |
| AB092 | D | Pulm |  | (0/4) | *Positive* | S | Positive | 13,35 |  | 254 (10.4) |
| AB115 | D | Pulm |  | (0/2) | *Positive* | S | Positive | 15,54 |  | 442 (20.3) |
| CF031 | D | Pulm |  | (0/5) | *Positive* | S | Positive | 10,12 |  | 335 (24.9) |
| CF052 | D | Pulm |  | (3/4) 1-9 | *Positive* | S | Indeterminate | 0,03 |  | 760 (16.2) |
| CF059 | D | Pulm |  | (0/8) | *Positive* | R* | Positive | 4,92 |  | 495 (22.7) |
| CF159 | P | Pulm |  | (1/4) <1 | *Négative* | - | Negative | 0,01 |  | 605 (19.6) |
| CF162 | D | Pulm |  | (0/5) | *Positive* | R | Positive | 11,99 |  | 471 (20.3) |
| CF181 | D | Pulm |  | (1/4) <1 | *Positive* | S | Positive | 18,1 |  | 465 (14.4) |
| HB031 | D | Pulm |  | (4/4) >100 | *Positive* | R** | Positive | 10,73 |  | 468 (22.5) |
| HB051 | D | Pulm |  | (0/9) | *Positive* | S | Positive | 1,36 |  | 348 (19.2) |
| KT012 | D | Pulm |  | (1/7) < 1 | *Positive* | S | Positive | 18,02 |  | 375 (24.5) |
| NS044 | D | Pulm |  | (0/4) | *Positive* | S | Positive | 2,54 |  | 490 (27.2) |
| NS106 | D | Pulm |  | (0/3) | *Positive* | R*** | Positive | 11,58 |  | 496 (23) |
| NS169 | D | Pulm |  | (0/1) | Negative | - | Positive | 4,97 |  | 342 (15.6) |
| NS191 | D | Pulm |  | (0/5) | *Positive* | S | Positive | 6,17 |  | 484 (21.2) |
| ST113 | D | Pulm |  | (0/3) | *Positive* | S | Positive | 8,64 |  | 432 (20.1) |

**Footnotes to Table 1A S:**

*AFB =acid fast bacilli smear; D: definitive; P: probable; Pos/total : number of positive samples/total number of samples; Max AFB/field: maximum of bacilli per field in positive samples

** positive: all with M*.* tuberculosis complex mycobacteria

ATB: antibiogram

NA: non avalaible

S: All antiTB drugs tested sensitive (Rifampicine, Streptomycine, INH, Ethambutol)

R: Resistance to at least one antiTB drug

R*: Ethambutol and Rifampicine Resistance

R**: INH and Rifampicine Resistance

R***: INH and Strepto Resistance
