# Supplementary material for: Quantiferon-TB Gold: Performance for Ruling out Active Tuberculosis in HIV-Infected Adults with High CD4 Count in Côte d'Ivoire, West Africa
Source: PLoS One. 2014 Oct 16;9(10):e107245. doi: 10.1371/journal.pone.0107245 (PMC4199568; doi:10.1371/journal.pone.0107245)
Supplement: Table S4 — Conversion and reversion of the QuantiFERON TB Gold in-tube test between Day-0 and Month-12, according to different thresholds. (DOCX) [file pone.0107245.s004.docx]

**Table 4S:** **Conversion and reversion of the QuantiFERON^®^ TB Gold test between Day-0 and Month-12, according to different thresholds**

|  | TB Antigen minus Nil Threshold (IU/ml) | |  | Patients | | | | |  |
| --- | --- | --- | --- | --- | --- | --- | --- | --- | --- |
|  | Baseline | Month-12 |  | Day-0 |  | Month-12 | | |  |
|  |  |  |  | n |  | n | % | 95% CI |  |
| Conversion | **< 0.35** | **≥ 0.35** |  | **261** |  | **24** | **9.2%** | **( 5.7 - 12.7 )** |  |
|  | < 0.35 | **≥** 0.70 |  | 261 |  | 16 | 6.1 % | ( 3.2 – 9.0 ) |  |
|  | < 0.35 | **≥** 0,35 and **≥**30% increase since baseline |  | 261 |  | 24 | 9.2% | ( 5.7 – 12.7 ) |  |
|  | < 0.35 | **≥** 0,35 and **≥** 0,35 IU/ml increase since baseline |  | 261 |  | 21 | 8.1% | ( 4.8 – 11.4 ) |  |
|  | < 0.35 | > 10 |  | 261 |  | 6 | 2.3% | (0.5 – 4.1) |  |
| Reversion | **≥ 0.35** | **< 0.35** |  | **167** |  | **24** | **14.4**% | **( 9.1 – 19.7 )** |  |
|  | **≥** 0.70 | < 0.35 |  | 146 |  | 16 | 11.0% | ( 5.9 – 16.0 ) |  |
|  | **≥** 0.35 | **≥** 30% decrease since baseline |  | 167 |  | 24 | 14.4% | ( 9.1 – 19.7 ) |  |
|  | **≥** 0.35 | **≥** 0,35 IU/ml decrease since baseline |  | 167 |  | 23 | 13.8% | ( 8.6 – 19.0 ) |  |
|  | > 10 | < 0.35 |  | 64 |  | 3 | 4.7% | (0.0 – 9.9) |  |
| No conversion | **<0.35** | **< 0.35** |  | **261** |  | **237** | **90.8%** | **(87.3 – 94.3)** |  |
| No reversion | **≥ 0.35** | **≥ 0.35** |  | **167** |  | **142** | **85.0%** | **(79.6 – 90.4)** |  |
| Other scenarios | Indeterminate | ≥ 0.35 |  | 2 |  | 0 | 0.0% | (0.0 – 0.0) |  |
|  | Indeterminate | < 0.35 |  | 2 |  | 1 | 50.0% | (0.0 - 100.0) |  |
|  | <0.35 | Indeterminate |  | 261 |  | 0 | 0.0% | (0.0 – 0.0) |  |
|  | >0.35 | Indeterminate |  | 167 |  | 1 | 0.6% | (0.0 – 1.8) |  |
|  | Indeterminate | Indeterminate |  | 2 |  | 1 | 50.0% | (0.0 - 100.0) |  |

Footnotes to table 2A:

CI: confidence interval;

n=number;

% percentage;

IU/ml : international units per millimeter
