# Supplementary material for: Quantiferon-TB Gold: Performance for Ruling out Active Tuberculosis in HIV-Infected Adults with High CD4 Count in Côte d'Ivoire, West Africa
Source: PLoS One. 2014 Oct 16;9(10):e107245. doi: 10.1371/journal.pone.0107245 (PMC4199568; doi:10.1371/journal.pone.0107245)
Supplement: Table S5 — Factors associated with conversion, defined as QTF <0,35UI/ml at baseline and QTFTB test >0,35UI/ml at Month-12, univariable analysis. (DOCX) [file pone.0107245.s005.docx]

**Table 5S: Factors associated with conversion, defined as QTF <0,35UI/ml at baseline and QTFTB test > 0,35UI/ml at Month-12**, **univariable analysis**

|  | **Univariable analysis** | | | |
| --- | --- | --- | --- | --- |
| **Variable** | **OR** | **CI _95%_** | | ***P*** |
| No ART between M0 –M12 (yes vs No) | 1.15 | (0.48- | 2.75) | 0.74 |
| INH Prophylaxis INH (Yes/no) | 0.87 | (0.37- | 2.01) | *0.74* |
| Hemoglobin (<10 g/dl  *vs* > 10 g/d) | 1.30 | (0.51- | 3.29) | *0.58* |
| Viral Load, log/10 ml (<5 *vs*  >5) | 1.53 | (0.58- | 4.00) | *0.39* |
| Age (18-40 vs >40 years old ) | 0.62 | (0.26- | 1.48) | *0.28* |
| WHO stage (stage 3 vs Stage 1-2) | 1.16 | (0.32- | 4.15) | *0.82* |
| Sex (Female vs male) | 0.91 | (0.32- | 2.58) | *0.87* |
| CD4 Count at inclusion, <500 *vs* > 500/mm3 | 0.47 | (0.19- | 1.16) | *0.10* |
| HbS Antigen (positive vs negative) | 0.89 | (0.19- | 4.03) | *0.88* |
| Delta CD4 count (M12-M0) <0 (yes vs No) | 1.25 | (0.52- | 2.98) | *0.62* |
| BMI (<25 vs > 25kg/mm2) | 0.71 | (0.25- | 1.98) | 0.52 |

**Footnotes to Table 3A S**

OR: odd ratio

CI _95%:_ Confidence interval

ART :Antiretroviral treatement

INH :Izoniazid

WHO: World Health Organisation

BMI: Body Mass Index
