# Supplementary material for: Quantiferon-TB Gold: Performance for Ruling out Active Tuberculosis in HIV-Infected Adults with High CD4 Count in Côte d'Ivoire, West Africa
Source: PLoS One. 2014 Oct 16;9(10):e107245. doi: 10.1371/journal.pone.0107245 (PMC4199568; doi:10.1371/journal.pone.0107245)
Supplement: Table S6 — Factors associated with reversion, defined as QTF >0,35UI/ml at baseline and QTFTB test <0,35UI/ml at Month-12, univariable and multivariable analysis. (DOCX) [file pone.0107245.s006.docx]

**Table 6S : Factors associated with reversion ,defined as QTF >0,35UI/ml at baseline and QTFTB test <0,35UI/ml at Month-12, univariable and multivariable analysis**

|  | **Univariable analysis** | | | | **Multivariable analysis, final model** | | | |
| --- | --- | --- | --- | --- | --- | --- | --- | --- |
| **Variable** | OR | CI _95%_ | | *P* | OR | CI _95%_ | | P |
| INH Prophylaxis INH (Yes/no) | 1.68 | (0.70- | 4.03) | *0.24* | - |  | - | - |
| Hemoglobin (<10 g/dl  *vs* > 10 g/d) | 0.62 | (0.19- | 1.93 ) | *0.41* | - |  | - | - |
| Viral Load, log/10 ml (<5 *vs*  >5) | 1.30 | (0.51- | 3.36 ) | *0.58* | - |  | - | - |
| CD4 Count at inclusion, <500 *vs* > 500/mm3 | 0.88 | (0.30- | 2.58 ) | *0.82* | - |  | - | - |
| Age (18-40 Years old vs >40 years old ) | 7.29 | (1.65- | 32.2) | *0.009* | 6.70 | (1.48 | 30.26) | *0.013* |
| WHO stage (stage 3vs Stage 1-2) | 0.47 | (0.10- | 2.16) | *0.33* | - |  | - | *-* |
| Sex (Female vs male) | 1.68 | (0.54- | 5.25 ) | *0.37* | - |  | - | *-* |
| HBS Antigen (positive vs negative) | 0.73 | (0.09- | 6.15) | *0.77* | - |  | - | *-* |
| Delta CD4 count (M12-M0) <0 (yes vs No) | 2.88 | (1.19- | 6.96 ) | *0.02* | - |  | - | *-* |
| No ART during the period (yes vs no) | 5.12 | (1.98- | 13.2 ) | *0.0007* | 4.78 | (1.81 | 12.62) | *0.001* |
| BMI ( >25kg/m2 vs < 25) | 1.31 | (0.48- | 3.59) | *0.59* | - |  | - | *-* |

**Footnotes to Table 4A S**

OR: odd ratio

CI Confidence interval

ART Antiretroviral treatement

INH Izoniazid

WHO World Health Organisation

BMI Body Mass Index
